# Amplifying youth voices: young people’s recommendations for policy and practice to enhance vaccine acceptability

**DOI:** 10.1186/s12913-024-11630-8

**Published:** 2024-11-18

**Authors:** Oluwaseyi Dolapo Somefun, Marisa Casale, Genevieve Haupt Ronnie, Joshua Sumankuuro, Olagoke Akintola, Chris Desmond, Lucie Cluver

**Affiliations:** 1 School of Public Health, University of the Western Cape, Bellville, Robert 11 Sobukwe Rd, Western Cape, 7535 South Africa; 2 Department of Social Policy and Intervention, University of Oxford, Barnett 13 House, Oxford, 14 3 UK; 3 Centre for Social Science Research, University of Cape Town, Cape Town, South Africa; 4 Department of Public Policy and Management, Faculty of Public Policy and Governance, Simon Diedong Dombo University of Business and Integrated Development Studies, Bamahu, Ghana; 5 School of Nursing, Paramedicine and Healthcare Sciences, Faculty of Science and Health, Charles Stuart University, Bathurst, NSW Australia; 6 School of Economics and Finance, University of the Witwatersrand, Johannesburg, South Africa; 7 Dept of Psychiatry and Mental Health, University of Cape Town, Cape Town, South Africa

**Keywords:** Vaccine acceptability, Young people, Africa, Policy, COVID-19 pandemic

## Abstract

**Background:**

The COVID-19 pandemic has underscored the need for increased vaccine availability and uptake, with vaccine hesitancy posing a significant barrier, particularly among young adults. Evidence from various countries highlight high levels of hesitancy among young people, necessitating targeted interventions. Engaging young adults as key stakeholders in shaping public health strategies is crucial, as their perspectives can enhance vaccine acceptance. This study aimed to assess the overall acceptability of the COVID-19 vaccine among young people and to explore the factors influencing their willingness or reluctance to be vaccinated now and in the future.

**Methods:**

This study used qualitative data from 165 young adults in Nigeria, South Africa, and Zambia, to explore their suggestions for policies and strategies aimed at enhancing the acceptance of the Covid-19 vaccination among their age group. Data collection involved focus groups and interviews that explored participants’ perceptions and recommendations regarding COVID-19 vaccination acceptability. Thematic analysis was used to analyse the data.

**Results:**

Thematic analysis identified several factors influencing vaccine acceptability among young people and suggested recommendations to improve it. The themes included developing targeted communication strategies for accurate vaccine information, offering alternative vaccination methods, promoting vaccine education in schools, and using trusted public figures to share accurate information.

**Conclusions:**

Persistent dissatisfaction with vaccine information dissemination underscores the need for more targeted communication strategies among young adults. Recommendations include developing non-injection vaccine options, incorporating vaccine education into school curricula and community programs, and leveraging influential public figures to build credibility. These insights are valuable for designing future programs to enhance vaccine acceptance among adolescents.

**Supplementary Information:**

The online version contains supplementary material available at 10.1186/s12913-024-11630-8.

## Introduction

The COVID-19 pandemic highlighted the need for rapid vaccine development, distribution, and equitable access across different regions and populations, to effectively control the virus. Despite proven efficacy in controlling the virus’s spread, achieving high vaccination rates remains a challenge. Rising vaccine hesitancy, now integrated into mainstream beliefs, threatens the success of current and future vaccination campaigns [1, 2] .

Vaccine hesitancy, characterized by delayed acceptance or outright refusal of available vaccines [3], is recognized by the World Health Organization (WHO) as a significant global health threat. Negative healthcare experiences and general distrust in government have cultivated a climate conducive to vaccine hesitancy across Africa [4]. The proliferation of misleading information regarding vaccine side-effects on social media further compounds this challenge [5, 6].

Regrettably, the effectiveness of COVID-19 vaccines in curbing the virus has not translated into a decline in global vaccine hesitancy [7]. The rapid development, approval, and distribution of COVID-19 vaccines have exacerbated pre-existing distrust and suspicion [3]. Consequently, regions historically grappling with healthcare access and equitable supplies now face a new obstacle - insufficient vaccine acceptance, both for COVID-19 and other diseases. Addressing these concerns and countering vaccine-related misinformation is imperative to safeguard the progress achieved in disease prevention through vaccination.

In particular, evidence from various parts of the world highlight the concerning prevalence of COVID-19 vaccine hesitancy among adolescents [8]. Evidence from a multi-country study conducted between July and December 2021 uncovered alarmingly high rates of COVID-19 vaccine hesitancy among adolescents in sub-Saharan African countries including Burkina Faso, Ethiopia, Ghana, Nigeria, and Tanzania [9]. This study found that concerns regarding the safety and efficacy of COVID-19 vaccines were primary drivers of vaccine hesitancy. Specifically, perceptions of inadequate safety and effectiveness were significant predictors of increased reluctance to get vaccinated. These findings underscore the urgent need for region-specific strategies to address vaccine hesitancy and its determinants among adolescents, considering the unique dynamics at play in Africa.

“Young people”^1^, encompassing adolescents and young adults, constitute a substantial portion of the global population, and their buy-in and engagement are crucial in effectively addressing public health crises. As the digital natives of today’s interconnected world, they possess unparalleled access to information and play a pivotal role in shaping societal attitudes and behaviours [10, 11]. In this context, young people should be considered key stakeholders. Recognizing this, it is essential to increase vaccine acceptability and uptake among young people and to ensure that their concerns, experiences, and recommendations are incorporated into policy and practice. Doing so creates the opportunity for young people to bring fresh perspectives and unique insights that can help inform policies and practices to increase vaccine uptake and acceptability [12].

Nevertheless, a limited number of studies document young people’s first-hand perspectives regarding the factors influencing low vaccine acceptability among themselves and their peers. Even fewer grant them the opportunity to directly put forward their suggestions on measures that policymakers and practitioners could take to make vaccination more acceptable in this population group. We believe that the absence of youth voices in policy recommendations has hindered comprehensive strategies to improve vaccine acceptability among young people.

Drawing from qualitative data collected with young adults in Nigeria, South Africa, and Zambia, this paper presents and discusses young people’s recommendations for policy and practice, to increase acceptability of the Covid-19 vaccine among young people, and more broadly address the unique challenges surrounding vaccine uptake in this age group. By exploring young people’s experiences, perspectives, and recommendations related to vaccination for Covid-19, we sought to better understand the drivers of low and high vaccine acceptability in this population group. Moreover, we wanted to know how young people themselves felt these factors could be addressed to increase vaccine acceptability and uptake among young people.

## Methodology

### Framework

**Fig. 1:**
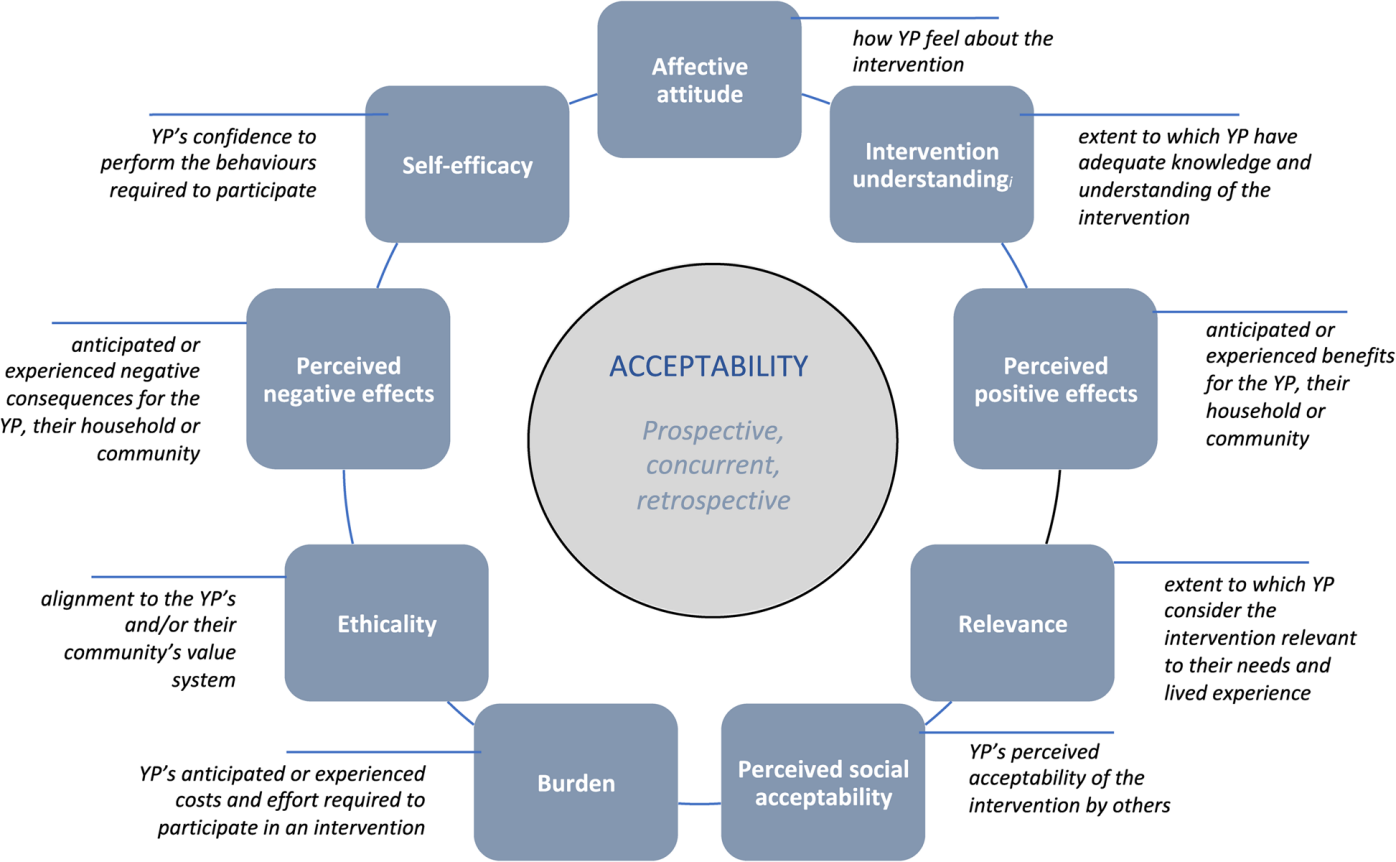
The accelerate framework for youth acceptability [13]

This exploratory qualitative study is part of a broader body of research, guided by the recently developed Accelerate *Framework for Young People’s Acceptability* [13] (Fig. 1). The framework informed the development of data collection tools and guided the process of data analysis. Acceptability is described as a complex concept that gauges how individuals involved in delivering or receiving a healthcare intervention perceive its appropriateness, considering cognitive and emotional responses [13, 14]. The *Accelerate Framework for Youth Acceptability* proposes nine elements of acceptability: affective attitude, intervention comprehension, perceived positive outcomes, relevance, perceived social acceptance, burden, ethical considerations, perceived negative effects, and self-efficacy. Figure 1 taken from [13], illustrates and defines each of these components.

### Sampling and data collection

We conducted 12 in-person focus groups and 38 remote interviews with 165 individuals, aged 15–24, from South Africa, Zambia, and Nigeria (see Table 1).

**Table 1:** Frequency distribution of participants

In-depth interviews				FGDs
		Gender		
Country	Number of participants	Female	Male	
South Africa	9	5	4	12
Nigeria	27	15	12	-
Zambia	2	2	-	-

We employed convenience sampling due to practical considerations to recruit participants, leveraging our networks and connections with organizations providing interventions and services to young adults. We partnered with 8 community-based organizations (CBOs) providing sexual and reproductive health and rights (SRHR) or skills building interventions for our target age group, of which three were based in South Africa, one in Zambia, and four in Nigeria. For in-person focus groups conducted in South Africa, CBO staff collaborated with researchers to directly enlist participants for focus groups. For all individual interviews (IDIs), CBO representatives contacted potential participants to gauge their interest in participating and being approached by researchers. Subsequently, researchers directly reached out to interested individuals. Utilizing lists of potential participants provided by the CBOs, researchers contacted them one by one until reaching the desired number of willing participants, striving for equal representation of both genders within the specified age bracket (15–24 years). This process was similar for recruiting participants in Nigeria and Zambia, although, in-depth interviews were conducted online with participants from these two countries.

During the research period, all authors were situated in South Africa, which posed logistical challenges for organizing group discussions in Nigeria and Zambia. Our use of a convenience sampling method, reliant on referrals from existing networks, further complicated the coordination of online focus groups with young participants. Given the diverse backgrounds of the participants—spanning Northern (East and West), South-Western Nigeria and Zambia—we chose to conduct in-depth interviews in these two countries. This approach enabled us to obtain detailed and nuanced insights, which were crucial for understanding the varied experiences and contexts of the participants.

Our sampling strategy was primarily influenced by considerations of accessibility and convenience, constrained by the limited available time and resources. Additionally, the support and willingness of community-based organizations (CBOs) to collaborate with us and facilitate recruitment were crucial. These CBOs, with their established networks in the communities, enabled us to effectively recruit participants within our limited timeframe and resources.

Data was collected by means of semi-structured topic guides for focus groups and interview guides for IDIs as employed in other studies focusing on young adults [15, 16]. (see supplementary file). Guided by our theoretical framework and reviews of the empirical literature on acceptability, we used two overarching approaches. The first approach consisted of more generic, open-ended questions. We asked young adults to assign a rating between 1 to 5 to the Covid-19 vaccine ‘intervention’, with 1 representing the lowest and 5 indicating the highest level of acceptance. Following this, participants were prompted to provide qualitative justifications for their assigned ratings and (for those who provided a score lower than 5) asked what could be done to improve their ratings. The second approach consisted of more specific questions, for each of the nine distinct components of our framework. For instance, in relation to the ‘intervention understanding’ component, we posed the following question: “What do you think could have been done to improve your understanding?” For other components like “relevance”, we asked; “What do you think could have been done to make it more relevant to you?” We therefore prompted young people to provide recommendations both to improve overall acceptability of the Covid-19 vaccines, through an open question, and to provide more specific recommendations on how to improve each of the nine specific components of acceptability represented in the *Accelerate* framework.

In-depth interviews lasted for a minimum of 60 min and were conducted in English in Nigeria and Zambia. Focus group discussions, conducted in the local language (Zulu), ranged from 1 to 2 h in duration. Two of the authors conducted all the in-depth interviews, while the focus group discussions were conducted by two bilingual field research assistants who were trained in engaging young adults in discussions, with oversight from the lead author. Two of the authors involved in data collection hold PhDs. One of these lead authors has over 20 years of field research experience, while the other has six years. Another team member who supported the interviews has about five years of extensive research and data collection experience. The focus group discussions were facilitated by two bilingual field research assistants, each with over 15 years of experience working with children, young people, and caregivers. These assistants also received rigorous ethics training. They were supervised by one of the lead authors, who ensured the quality and consistency of the discussions. The entire team, including the field research assistants, was well-trained in data collection methodologies through various training sessions and workshops.In-depth interviews were recorded and transcribed by the authors; focus group discussions were transcribed, translated into English, and subsequently quality controlled by a second translator.

### Rigour

Trustworthiness was ensured through an iterative process that involved multiple discussions of findings among the authors. We consistently identified and addressed quotes that contradicted the emerging themes and employed selective member checking throughout the process. The initial findings were reviewed and openly discussed among the authors, allowing for ongoing refinement and validation of the results [17]. Detailed documentation of the data collection procedures further supported transparency and allowed for comprehensive review. These strategies collectively contributed to the robustness and credibility of the study’s results.

Ethical approval for this study was obtained from the Biomedical Research Ethics Committee of the University of the Western Cape (BM21/10/39). We obtained written consent from all participants, with additional consent obtained from parents or guardians for participants under 18 years of age. Consent forms were translated into the relevant local languages and explained verbally to participants. As a token of appreciation for their involvement in the research, IDI participants received airtime compensation, while the FGD participants were provided with snacks and a hot meal, and reimbursed for their transport expenses.

### Data analysis

We analysed the data collaboratively using Dedoose software, enabling research team members to work online simultaneously from different geographical locations. Thematic analysis was the primary method utilized, employing an iterative process that combined deductive and inductive approaches (Corbin & Strauss, 1990; Neuman, 2006; Ritchie & Lewis, 2006). Four authors conducted the data analysis, collaborating closely with bilingual interviewers who led focus groups in KwaZulu-Natal. Two of these authors also collected data in Nigeria and South Africa.

Thematic data analysis for this paper was conducted in two stages. Initially, a broad analytical framework was established, drawing upon the nine components of the acceptability theoretical framework and including a tenth primary theme and code to broadly capture recommendations for policy and practice provided by young individuals. Data pertaining to multiple overarching themes could be coded multiple times and we noted that, as expected, there was some overlap. Subsequently, we carried out a secondary level of thematic analysis, specifically for the data pertaining to the overarching theme of young people’s recommendations. This allowed us to identify and organize multiple layers of subthemes.

The collaborative analysis processes commenced with the four researchers (and co-authors) collectively reading through all transcripts without applying coding, to familiarize themselves with the data and highlight any noteworthy points. Subsequently, each researcher independently coded three transcripts, ensuring that at least one was from a focus group session. Initial themes were derived from the transcript content, employing language similar to that used by respondents, while particular attention was paid to the treatment and presentation of these themes. The team then met virtually to compare and discuss their initial set of codes identified and generate an initial analytical framework. Main themes and subthemes identified were implemented as a code tree using Dedoose software, with agreed-upon definitions for each code to aid coders. Subsequently, the researchers split the remaining transcripts for coding. Over the course of several months, regular virtual meetings were held to identify, refine, and review themes, address challenges, and iteratively improve the analytical framework.

### Findings

Two overarching themes emerged from participants’ recommendations: “Healthcare-related recommendations” and “Information and Training recommendations”. Further, for each of these two broad themes, several subthemes were identified; these are represented in Table 2.

**Table 2:** Themes and sub-themes

Main Theme	Sub-theme
Healthcare-related recommendations	Examining Non-Injectable Vaccine Approaches
	Optimizing Vaccine Safety and Accountability
	Developing Youth-friendly Health Systems
	Driving innovative Strategies to enhance Accessibility
Information and Training recommendations	Using public Figures as Vaccine Role Models
	Utilizing media for Vaccine Awareness
	Fostering school-Based Education and Teacher Training
	Promoting engagement through Community Campaigns

### Theme 1: healthcare-related

The healthcare-related theme includes the perspectives and recommendations of young participants regarding the healthcare system’s role in vaccine acceptability. This theme is elucidated through the following subthemes: “Examining Non-Injectable Vaccine Approaches”, “Optimizing Vaccine Safety and Accountability”, “Developing Youth-friendly Health Systems”, and “Driving innovative Strategies to enhance Accessibility”.

### Examining non-injectable vaccine approaches

Young participants strongly advocated for alternatives to vaccines in the healthcare system. Motivated by their fear of injections and a desire for greater convenience, youth participants asserted that alternative methods of vaccine administration should be actively considered and put into practice. Their specific recommendations include exploring and developing non-injectable approaches, such as oral medications or enemas, to cater to individuals with needle fear or specific cultural and religious beliefs that could clash with the use of injectables. As explained by two female focus group participants:

“*I am afraid of injection*,* so for me*,* it would be better if there was something that can be taken orally*,* something you can drink” (F*,* FGD*, South Africa*).*

*They can develop medicine or pills that can be consumed. Also*,* for the convenience of those that have certain beliefs as we had mentioned earlier. They can use an enema to fill their bodies with the vaccine. In essence*,* I am trying to give possible examples of how people who are scared of needles can be accommodated (F*,* FGD*, South Africa*).*

### Optimizing vaccine safety and accountability

Young adults also highlighted the importance of assessing individual health status before administering vaccines, to avoid adverse interactions with existing medical conditions and treatments. They believed that situations where vaccines were mistakenly blamed for pre-existing illnesses or ongoing treatments could be avoided. For instance, one youth stated: “*Before people get vaccinated their medical history check-up should be prioritized. To avoid a situation where the vaccination is blamed over the sickness they have been treating for quite sometime*.” (F, FGD, South Africa).

Another youth in the FGD added: “I *was saying that you should allow them to ask you questions about your health condition so that they will know the risks of injecting you with a certain vaccine*,* in case you have some illness and how you might be affected*.” (F, FGD, South Africa).

However, contrasting perspectives also emerged. Another individual within the same focus group expressed a different stance: “*They should not ask you too many questions. They must just stick to what they came to do*,* not ask things like ‘are you HIV positive?’ or anything like that*,* or ‘what health issues do you have?’. You have come to get vaccinated*,* not to be interrogated*.” (F, FGD, South Africa).

### Developing youth-friendly health systems

Youth also emphasized the importance of comprehensive training for healthcare providers, to ensure they possessed the necessary skills and knowledge to effectively treat their clients. Participants highlighted the need for healthcare professionals to undergo step-by-step training to enhance their ability to provide respectful and empathetic care. They suggested that, by fostering respectful communication, healthcare providers could create a more welcoming and comfortable environment for their clients. For instance, one of the IDI participants said: “*Stepdown training can be carried out. Yes*,* to know how to be able to treat their clients. Not speak rude”. (M*,* IDI*,* Nigeria).*

Another young person alluded to the need for more accommodating healthcare structures. They highlighted the need for a systemic shift towards inclusivity, streamlining medical facilities for the ease and engagement of the youth demographic: “*Hmm and secondly*,* maybe not shove us in a corner*,* because here we have youth friendly corners*,* we do not have a user friendly system. Like we have youth friendly corners so we could have a youth friendly system. it would be easier for us even to go. Not only concerning Covid but even for our health generally*,* it would be easier for us to go to medical facilities*” (F, IDI, Zambia).

### Driving innovative strategies to enhance accessibility

While highlighting the importance of fostering inclusivity and overcoming barriers to vaccination, participants suggested innovative strategies to enhance accessibility. One suggested approach was the implementation of mobile vaccination sites and community-based outreach programs. These suggestions reflected a collective desire to address mobility issues and transportation challenges faced by individuals, particularly those who could not afford transport costs. For instance, one FGD participant expressed: *“I think they should come to the community and vaccinate people there”* (F, FGD, South Africa), while another suggested the use of mobile clinics: “*They must introduce mobile clinics*,* so that people don’t find themselves having to travel long distances to vaccinate”* (M, FGD South Africa).

Young people also suggested household visits to people who were immobile because of age, illness or disability. For instance, one FGD participant made the following recommendation: “*If there are people who go around the community and ask for those who want to vaccinate and do them in their household… because we have grannies that we stay with that would like to be vaccinated*,* but they can’t since their legs can’t function properly*,* and there’s the transport issue” (F*,* FGD* South Africa*).*

### Theme 2: information and communication

The overarching theme of ‘information and communication’ highlights the influence of educational initiatives and the accessibility of accurate, reliable information on the acceptance of vaccinations among participants. Young people emphasized the importance of raising vaccine awareness in schools, community centres, and public forums, to empower individuals in making informed decisions about their health and well-being. Sub-themes identified were: “Using public figures as vaccine role models”, “Utilizing media for vaccine awareness”, “Fostering school-based education and teacher training” and “Promoting engagement through community campaigns”.

### Using public figures as vaccine role models

During the focus group discussions, numerous youth participants emphasized the significance of prominent figures, such as the country’s president, publicly receiving vaccines to bolster public trust. One of the male FGD participants mentioned: “*It would be nice if the president will be shown on television while he is receiving a vaccine so that we can see for ourselves whether he is given the same thing that everyone else receives*” (M, FGD, South Africa).

Other young adults expressed a desire to witness transparency in the vaccination process, with a few participants suggesting showcasing events on television. Specifically, they referenced President Cyril Ramaphosa’s televised addresses as a blueprint for disseminating accurate information. Some of the participants proposed a model where representatives from entities producing and administering the vaccine, such as Johnson & Johnson, would use national broadcasting platforms to inform the public.

### Utilizing media for vaccine awareness

Harnessing both conventional and social media emerged as a strategy considered crucial by young people to enhance vaccine acceptability. Participants emphasized the potential of television and news platforms, suggesting the incorporation of dedicated time within established news broadcasts for vaccine information sessions: “*We have a news bulletin at 7:00 pm. So*,* they can use 15 minutes of the time for the news and then allocate some minutes for the weather forecast*,* and then spend 10 minutes of the time explaining the vaccine” (F*,* FGD*, South Africa*).*

Participants also highlighted the critical role of social media in raising awareness. Suggestions from in-depth interviews underscored the ease of disseminating information through platforms such as Facebook, Twitter, and WhatsApp, that many young people were habitually accessing. Participants emphasized the importance of user-friendly content and detailed information. For example, one young person suggested the use of paid advertisements on Facebook to reach the youth effectively: *“Let that information be friendly*,* maybe on Facebook*,* on Twitter*,* on WhatsApp pages. Give us detailed information*,* okay?” (M*,* IDI*,* Nigeria).*

Another said: *“… most of the youth spend their time on Facebook*,* there are paid adverts on Facebook that… you should go and take it and give us detailed instruction on how it is - if you take the vaccine*,* you will not be affected by Covid” (M*,* IDI*,* Nigeria).*

However, other participants expressed reservations about the unregulated nature of social media, emphasizing the need for trustworthy and well-informed sources to counterbalance potential misinformation online. As explained by an FGD participant: “*The problem with social media*,* whatever is posted there is not regulated. Anyone can just forward their untested views. But if they [government or other authorities in power] can send someone who is well trained or informed*,* that will be much appreciated. Someone we can trust*” (F, FGD South Africa).

### Fostering school-based education and teacher training

Participants suggested visiting schools and organizing educational sessions as proactive measures to enhance vaccine awareness. They highlighted that this strategy could involve training teachers to lead specialized COVID-19 and broader vaccination classes, that could emphasize effective information dissemination and address potential side effects. As indicated by a FGD participant: “*Perhaps*,* especially in schools*,* they should provide us with teachers who can take us for Covid-19 and vaccine classes. They should elaborate*,* especially on the side-effects*,* etc. The government should send out people knowledgeable about COVID-19 related issues. Perhaps by doing that*,* some may change their attitude towards the vaccine*” (F, FGD South Africa).

### Promoting engagement through community campaigns

Participants also emphasized the need for well-targeted public campaigns designed to educate individuals about vaccines. This included developing campaigns with a specific focus on vaccine education and awareness, that adopt a door-to-door approach to reach communities. As emphasized by an FGD participant: “*There should be campaigns that will be developed specifically to educate them [individuals] about the vaccine*” (F, FGD, South Africa). Another participant highlighted the importance of reaching out to people within the community and providing them with information about the vaccine: “*They (government) can even do a door-to-door campaign and teach people around the community about the vaccine*” (F, FGD, South Africa).

Recognizing the pivotal role of community involvement in increasing vaccine acceptance, some young people recommended that vaccine providers take proactive steps to connect and partner with local communities. This could involve utilizing community halls as information centres and forging partnerships with local representatives, such as councillors, to facilitate easier access to vaccination services. For example, a few FGD participants elaborated on this perspective: “*People who are in charge of the vaccine should come to the community and use community halls. They should get in contact with councillors in order to get close to the people*” (F, FGD, South Africa).

## Discussion

This study aimed to provide a direct voice to young people from Nigeria, South Africa, and Zambia, to allow them to make recommendations for policymakers and practitioners to increase Covid-19 vaccine acceptability. Our findings highlight how the swift development and distribution of COVID-19 vaccines may have intensified existing concerns around vaccines among adolescents and young adults. Vaccine hesitancy among these young people has been influenced by factors such as fear and unfamiliarity. Regardless of these challenges, young people have emerged as key stakeholders with valuable insights that can inform strategies to enhance vaccine acceptance. Notably, the recommendations provided by young people were consistent across the three countries, emphasizing the need for tailored communication strategies, educational initiatives, and innovative vaccine delivery methods, despite the cultural and contextual differences. This consistency underscores the universal nature of their concerns and the potential for common approaches to address vaccine hesitancy effectively.

We believe that our findings can be applied in two ways: first, to inform the design of tailored interventions that better resonate with young people’s desires and needs, paving the way for increased vaccine uptake and acceptability; second, to highlight areas where young people may need further information and engagement, to better understand some of the broader issues and why some of their recommendations may not be feasible in the short or longer term.

The pain associated with vaccine injections was described a source of distress for some participants and this has been highlighted in other studies [18–20]. The fervent advocacy among young participants for non-injectable vaccine approaches highlights crucial policy considerations for healthcare system improvements. To address their shared fear of injections and enhance convenience, policy initiatives should prioritize research and development funding for non-injectable methods, fostering innovation in vaccine delivery approaches such as oral medications and enemas. Microneedles (MNs) may offer a potential resolution to tackle these issues. A few recent studies have concluded that microneedles (MNs) are poised to serve as a viable substitute for existing vaccination methods [21–23]. However, while these non-injectable vaccine delivery methods offer potential benefits in terms of convenience and acceptability, prioritizing their development and implementation in resource-constrained settings in many countries may face significant challenges related to cost, infrastructure, cultural acceptance, regulatory hurdles, and competing healthcare priorities. Therefore, a more pragmatic approach that balances innovation with the urgent needs of the population may be warranted. More immediate measures could include dedicated vaccination clinics or mobile units, equipped with trained staff who can improve convenience and reduce anxiety associated with vaccination visits. This approach could also incorporate youth-friendly strategies and mobile structures, aligning with recent findings that highlight the importance of accessible and welcoming environments for younger demographics, thus ensuring inclusivity and effectiveness across all age groups.

Education and awareness stood out as a central theme among the recommendations for enhancing vaccine acceptance. Participants recognized the crucial role of elevating vaccine awareness to empower individuals in making well-informed decisions about their health. Their perspectives underscored the importance of integrating accurate information across diverse settings, including schools, community centres and public forums. Our findings indicate the importance of integrating comprehensive vaccine education into existing life skills programs within schools. Drawing from documented successes in South Africa [24] and other settings [25, 26], this integration could be seamlessly incorporated into current school curricula or life skills initiatives. Such an approach necessitates the development of age-appropriate educational materials and curricula that effectively convey the significance, safety, and efficacy of vaccines. Additionally, crafting health promotion messages tailored to specific subgroups can leverage data from reputable sources like the Africa Centres for Disease Control and Prevention, WHO, and national evidence-based repositories.

Visible leadership influence and representation emerged as a key theme. Participants stressed the importance of prominent figures, like the president, receiving vaccines on television to bolster public trust. This aligns with Erdogan’s research on celebrity endorsement [27]. Parallels were drawn with televised addresses by President Cyril Ramaphosa, highlighting the potential to provide accurate information and enhance credibility. Governments should collaborate with influential figures, including political leaders and celebrities, in public vaccination events. Utilizing ‘non-traditional’ messengers like celebrities or religious leaders and traditional rulers may be more impactful among youth [28]. Some studies have shown the positive association between social media influencers and vaccine awareness [29]. In Uganda, Bobi Wine’s “Everyone” initiative raised awareness of avoidable maternal fatalities, while his “Corona Virus Alert” campaign emphasized individual actions in combating the virus [28]. Programs could bring vaccine information directly to households, ensuring localized and accessible dissemination. This aligns with reaching diverse communities and addressing concerns at the grassroots level. Leveraging local influencers, community leaders, and trusted voices in a culturally sensitive manner is essential. Collaborating with religious institutions during congregational gatherings, where religious leaders play a pivotal role, can effectively convey vaccine importance within faith and community values [30, 31]. In India, religious leaders’ involvement promoted positive vaccine messages, enhancing trust and decreasing hesitancy [32]. However, policymakers must acknowledge and address potential challenges, such as differing views on vaccination within certain religious groups, with sensitivity and cultural competence.

Our research findings also highlight the importance of adopting a comprehensive and strategic approach to utilizing social and traditional media in order to enhance vaccine awareness among young people. This aligns with existing literature [33, 34]. Policymakers need to acknowledge the wide array of viewpoints shared by young individuals on social media platforms regarding vaccine effectiveness, reliability, and possible drawbacks. By understanding and engaging with these diverse perspectives, policymakers can tailor communication strategies that effectively address the concerns and preferences of young people, thus fostering greater vaccine acceptance and uptake. To address this, governments should harness widely used online social platforms like WhatsApp and Twitter to disseminate information on the advantages of COVID-19 vaccination. Thus, crafting public health campaign materials tailored to specific social media platforms and their users, incorporating emotive language and imagery prevalent in social media, can effectively enhance COVID-19 vaccine awareness [35]. Our findings suggest that employing tactics of peer influence can enhance vaccination acceptance rates. For instance, initiatives like UNICEF Nigeria’s “Give It A Shot” campaign exemplify this approach, wherein youths are educated about COVID-19 vaccines and encouraged to share this information with their peers, thereby fostering a culture of informed decision-making regarding vaccination [36].

Traditional media platforms can also serve as essential conduits for garnering public support for COVID-19 vaccinations and reaching segments of the population with limited access to or interest in digital media platforms [37]. This category encompasses television, radio, newspapers, magazines, medical journals, books, and pamphlets, all of which were prevalent before the rise of digital media and continue to hold significant ways in reaching populations in several African countries. The influence of media messaging and framing for vaccination programs has been extensively discussed elsewhere [38]. Policymakers can explore partnerships with trusted and widely consumed news outlets to secure prime-time slots for broadcasting reliable and informative content on the advantages of vaccination. The use of social and traditional media strategies recognizes the evolving media landscape and the importance of reaching diverse audiences through both online and traditional channels.

The participants’ recognition of the dual nature of social media, acknowledging its power in disseminating information but also expressing concerns about misinformation, highlights a policy imperative. They highlighted that policymakers should consider measures to regulate and monitor vaccine-related information on social media platforms. This may involve collaborating with tech companies to ensure the reliability of information and promote the involvement of credible and trained sources in disseminating vaccine-related content [39]. Nevertheless, it is essential to recognize the complexities involved. Tech companies operate on a global scale, each with its own policies, priorities, and challenges. Expecting uniform cooperation across all platforms may be unrealistic. Moreover, the definition of “reliable” information may vary, leading to potential conflicts over content moderation. Furthermore, overly stringent measures could erode public trust in both the government and social media platforms. Users may perceive such actions as paternalistic or authoritarian, leading to increased skepticism and resistance to public health initiatives. Building trust and promoting critical thinking skills among the population may be more effective in the long run than top-down regulation.

The policy implications arising from other findings also underscore the significance of in-person expert engagement in addressing vaccine-related concerns. Policymakers should consider initiatives that facilitate direct interactions between healthcare experts and individuals to enhance understanding, dispel misinformation, and motivate informed decision-making. Recognizing the value of personal communication, policymakers may explore strategies to integrate expert-led sessions into existing community outreach programs or healthcare initiatives. These strategies could involve identifying NGOs already engaging with young people and empowering them with messages on the vaccines, common concerns, and effective communication strategies.

Although our study focuses on COVID-19 vaccination, many of our findings are also applicable to future broader vaccination programs. Addressing the issue of “information overload” and conflicting messages from various sources with more targeted and tailored communication strategies is essential to effectively communicate relevant vaccine information for all types of vaccines, both for infectious disease control and routine immunizations. Our findings underscore the urgency of policy interventions to address injection fear, implement school-based education programs, and utilize visible leadership influence alongside diverse media channels for accurate vaccine information dissemination. Embracing these policy implications is crucial for developing inclusive, culturally sensitive vaccination approaches that resonate with diverse youth perspectives.

### Limitations

Since this is qualitative research, conducted through convenience sampling, our findings are not generalizable to the broader young population in Africa or even within the specific research areas. The use of convenience sampling means that the participants were not randomly selected, which could have introduced selection bias and limited the diversity of viewpoints. This method may not fully capture the range of experiences and perspectives within the broader young population. Additionally, the researchers’ social, cultural, and personal perspectives may have shaped the study’s approach, participant selection, and interpretation of the results, as is common in qualitative research.

## Conclusion

This study focuses on recommendations for vaccine uptake from young people in Nigeria, South Africa and Zambia. Young people often face different challenges and influences to older adults, such as increased exposure to social media, peer pressure, and differing levels of trust in health authorities. Their concerns and attitudes towards vaccines may be shaped by these factors, making it essential to address their specific needs and preferences through effective public health strategies. This study offers valuable insights into the challenges young people in Nigeria, South Africa, and Zambia face regarding COVID-19 vaccine acceptance, revealing significant vaccine hesitancy driven by fear and unfamiliarity. The findings underscore the importance of addressing these concerns through targeted interventions and comprehensive education. To enhance vaccine uptake, we recommend developing non-injection vaccine methods to provide more options, integrating vaccine education into school curricula and community programs, and leveraging influential public figures to build credibility. Additionally, optimizing media strategies to disseminate reliable information and combat misinformation, along with facilitating direct interactions between healthcare experts and young people, are crucial. These steps will help create a more inclusive and effective vaccination approach that will be more acceptable to young people.

## Supplementary Information

Supplementary Material 1.

## Data Availability

Data is provided within the manuscript or supplementary information files.

## Footnotes

1 In accordance with the classifications provided by the World Health Organization, the category of young individuals (ages 10–24) encompasses both adolescents (ages 10–19) and young adults (ages 15–24) as defined by Blum and Nelson-Mmari in 2004. For this paper, we will use the terms ‘young people’ and ‘young adults’ interchangeably to refer to this group.
