# Supplementary material for: Amplifying youth voices: young people’s recommendations for policy and practice to enhance vaccine acceptability
Source: BMC Health Serv Res. 2024 Nov 18;24:1425. doi: 10.1186/s12913-024-11630-8 (PMC11571648; doi:10.1186/s12913-024-11630-8)
Supplement: Supplementary file 1 — Supplementary Material 1. [file 12913_2024_11630_MOESM1_ESM.docx]

**Interview Guide for IDIs and FGDs**

**_______________________________________________________**

**Verbal Consent**

1. Have you seen the information sheet, and do you understand it?
2. Do you have any questions for me?
3. Do you consent for this interview?

***GENERAL DEMOGRAPHIC INFORMATION***

***(****to be collected for each participant separately, before the interview or FGD)*

**INSTRUMENT:** Interview guide on Covid-19 vaccine acceptability intervention

*Section 1: overall experience and opinions of the Covid-19 vaccine*

1a. Do you know that countries in Africa and across the world are starting to roll out vaccines for the Covid-19 virus to adults 12+ years of age and that the vaccine may soon be available even for younger adolescents? Since this is a really new intervention we want to understand what people think about it. No one knows much about what young people like you think about the COVID-19 vaccine and we think it is really important to know this. We’d really love to know what you think! Remember, feel free to tell us what you think and feel; there are no right or wrong answers.

1b. Have you and your peers been vaccinated? Why/why not? (*If answer is ‘no’)* Are you willing to be vaccinated? Would you recommend that others get vaccinated? Your peers for example? Your caregiver? Why/why not?

*If it is clear that the respondent is not willing to receive the vaccine or is hesitant:* I understand and respect that you may be uncertain about getting vaccinated. Is there anything that the part of you that would consider getting vaccinated would like or need for support? Do you think anything could make you change your mind?

2a. We are now going to ask you some more specific questions about what you know and think about this Covid-19 vaccine intervention:

1. When you think about getting vaccinated what is your first or overall feeling? (*probes: Good, bad? Do you like the idea?).* What do you think could make you feel more positive towards the vaccine?
2. Can you tell me how easy you think it was/would be for you to get vaccinated (*e.g. get to a vaccination site, have the vaccination*)? Did/does anything in particular make it hard for you? What do you think could be done to support you and other young people like yourself to get vaccinated easily?
3. Can you tell me about the costs you had/have to pay, or efforts you had/have to make to get vaccinated (*e.g. giving something up to participate, walking a long way to get to a vaccination site, stress related to this*)? Does this seem like a lot to you? Does it seem worth it? What do you think can be done to reduce these costs and efforts for you and other young people like yourself?
4. Can you tell me whether you had/have a good understanding of the Covid-19 vaccine, what it is and how it works? Do you feel you had/have been provided with sufficient information about the vaccine? What do you think can be done to improve understanding for you and other young people like yourself?
5. Can you tell me whether you think the vaccine works to prevent people from contracting and passing on Covid-19? Do you think getting vaccinated had/may have other positive effects for you, your family or your community? What do you think can be done to make it more effective and beneficial?
6. Can you tell me about any negative effects you think the vaccine could have for you? Do you think it could have any negative effects for your family and community? What do you think can be done to avoid or minimize these effects?
7. Can you tell me whether getting the Covid-19 vaccine was/is a priority in your life? Do you think it is something you and other young people like yourself need? Why/why not?
8. Can you tell me whether you feel that getting vaccinated fits with your value system? Is it in line with the values of your community? (*Example probes: are you concerned that it may conflict with religious, cultural or social beliefs and practices? is there something else that you take to prevent COVID-19 that isn’t a vaccine?*). Do you think anything can be done to make this vaccination intervention a better fit with your values?
9. Do you think that you getting the Covid-19 vaccine was/would be acceptable to the people in your life that are important to you, e.g. your partner, peers or caregivers? Do you think anything can be done to make the vaccine more acceptable to them?
10. Is there anything important that I have not asked you about, that affects your opinion of the Covid-19 vaccine and decision to be vaccinated? Can you tell me about it?

*Session closure*

Thank you so much for taking the time to participate in our research! You have provided us with very useful information which will help us understand more about what may affect young people’s decisions to get the Covid-19 vaccine and participate in other types of interventions. This is important for us to understand so that we can support – and help those running interventions support - the health and wellbeing of adolescents and young people!
